# Modeling of Nitrogen in River Water Using a Detailed and a Simplified Model

**DOI:** 10.1100/tsw.2001.351

**Published:** 2001-11-27

**Authors:** Mona Radwan, Alaa El-Sadek, Patrick Willems, Jan Feyen, Jean Berlamont

**Affiliations:** K.U. Leuven, Hydraulics Laboratory, Faculty of Engineering, Heverlee, Belgium

**Keywords:** ammonium, nitrate, water quality modeling, simplified model, sensitivity analysis

## Abstract

To model catchment surface water quantity and quality, different model types are available. They vary from detailed physically based models to simplified conceptual and empirical models. The most appropriate model type for a certain application depends on the project objectives and the data availability. The detailed models are very useful for short-term simulations of representative events. They cannot be used for long-term statistical information or as a management tool. For those purposes, more simplified (conceptual or meta-) models must be used. In this study, nitrogen dynamics are modeled in a river in Flanders. Nitrogen sources from agricultural leaching and domestic point sources are considered. Based on this input, concentrations of ammonium (NH_4_-N) and nitrate (NO_3_-N) in the river water are modeled in MIKE 11 by taking into consideration advection and dispersion and the most important biological and chemical processes. Model calibration was done on the basis of available measured water quality data. To this detailed model, a more simplified model was calibrated with the objective to more easily yield long-term simulation results that can be used in a statistical analysis. The results show that the conceptual simplified model is 1800 times faster than the MIKE 11 model. Moreover the two models have almost the same accuracy. The detailed models are recommended for short-term simulations unless there are enough data for model input and model parameters. The conceptual simplified model is recommended for long-term simulations.

### INTRODUCTION

Planning and management activities require the assessment of hydraulic and water quality conditions often beyond the range of observed field data. In this context both hydraulic and water quality models must be formulated that are general enough to (1) describe observed conditions and (2) predict planning scenarios that may be substantially different from observed conditions. In stream water pollution control, the main objective is to assess whether the system complies with the maximum pollutant releases, allowed from point and nonpoint source pollution, so that pollutant levels in the receiving streams meet water quality standards. Water quality models for in-stream water pollution control have been calibrated and verified with data collected prior to model development during surveys designed to check basin-wide water quality for regulatory compliance[1]. These data are typically inadequate for the following reasons:

- Many key water quality constituents or inputs are not measured because the purpose of the data collection is a general survey of water quality conditions in the stream system and not the development of a water quality model.
- There is tendency to sample certain water quality constituents because they are easy to sample, not because they increase knowledge of key water quality processes[2].
- The frequency of data collection is usually insufficient.

These inadequacies force water quality modelers to make weakly supported assumptions regarding model parameters or inputs and to use simplified model descriptions that require less input and parameters. Thus model prediction uncertainty increases and decision making for water pollution control is adversely affected.

In this study, the sensitivity is analyzed for two water quality models that are different in the level of complexity of the modeled processes. The evaluation is based on the following two aspects: the accuracy of the model results and the model speed.

### NITROGEN CYCLE IN RIVER WATER

All processes in the nitrogen cycle are summerized in a schematic way in Fig. 1. Two of the processes (ammonification and nitrification) convert gaseous nitrogen into usable chemical forms. The third process (denitrification) converts fixed nitrogen back to the unusable gaseous nitrogen state[3].

- **Ammonification** is a one-way reaction in which organisms break down amino acids and produce ammonia ($NH_3$). When BOD (biochemical oxygen demand) is modeled for the surface water, this process makes a clear correlation between the BOD decay process and the $NH_3$ production.
- **Nitrification** is the process in which ammonia is oxidized to nitrite and nitrate, yielding energy for decomposer organisms. Two groups of microorganisms are involved in nitrification. *Nitrosomonas* oxidizes ammonia to nitrite and water. Subsequently, *Nitrobacter* oxidizes the nitrite ions to nitrate.
- **Denitrification** is the process in which nitrates are reduced to gaseous nitrogen. This process is used by facultative anaerobes. These organisms flourish in an aerobic environment but are also capable of breaking down oxygen-containing compounds (e.g. $NO_3^-$) to obtain oxygen in an anoxic environment. Examples include fungi and the bacteria *Pseudomonas*[3].

In the soil, soil nitrate concentrations will vary seasonally with temperature and moisture levels. In temperate zones, fall and winter rains thoroughly remove all nitrates from the soil. No nitrate is naturally added to the soil during the late fall and winter because the cold weather prohibits mineralization and nitrification processes. During the spring and summer, the increased nitrogen-fixing activity of organisms and the addition of fertilizer cause the concentration of nitrates in the soil to steadily increase. Most of this nitrate is absorbed by plants. Thus the removal of crops in the fall increases the chances for large flushes of nitrate from the soil to water bodies. Some leaching may occur in the spring if crops are not established well enough to absorb the nitrogen[4].

#### Sources of Nitrogen in River Water

##### *Nitrate*

###### *Nonpoint Source*

- Agriculture: primary agricultural sources of nitrate include livestock excrement (from barnyards, pastures, rangeland, feedlots, and uncontrolled manure storage areas); nitrogenous fertilizers; irrigation return flows; and decomposing plant debris[5].
- Residential and urban: primary residential sources of nitrate include nitrogenous fertilizer used on lawn and garden, leaky

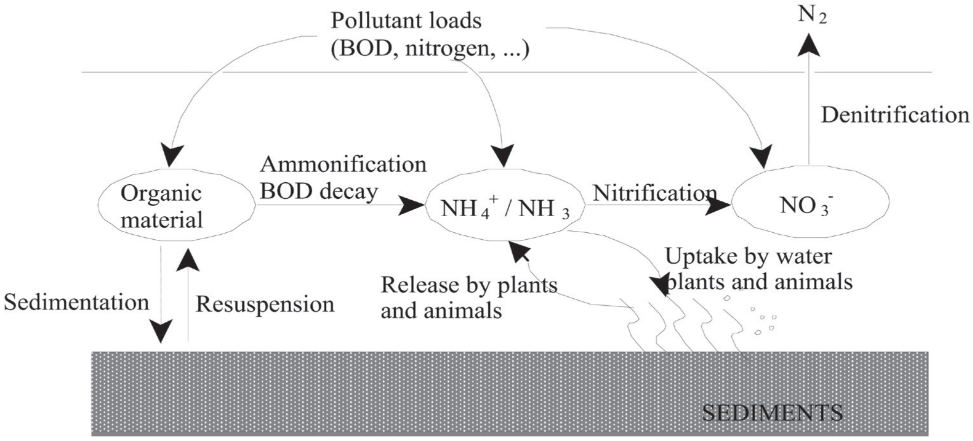

**FIGURE 1.** Processes related to the modeling of nitrogen ($NH_4^+$ / $NH_3$, $NO_3^-$).

on-site wastewater disposal/septic systems, and domestic pet excreta.

- Other: the combustion of fossil fuels, industrial and agricultural discharges of nitrogen-containing gases, aerosols, and airborne particles contribute to the atmospheric nitrogen load. Evidence suggests that the atmospheric deposition of nitrogen in water bodies (directly and via rainfall) constitutes a large portion of total nitrogenous inputs to estuarine and marine systems and a somewhat lesser portion of total nitrogen inputs to freshwater systems[6]. Additional nitrate sources include excreta both from wild animals in the surrounding watersheds, excreta from wildfowl congregating on the water body, and boats that discharge raw sewage overboard.

#### *Point Source*

- Industries that use nitrates in manufacturing may release nitrate in the effluent water. Nitrate is used in the following processes: meat curing, production of fertilizer, explosives, glass, heat-transfer fluid, and heat-storage medium for solar-heating applications[7]. Additional nitrates may be contributed by sewage treatment systems and combined sewer overflows (during high flow periods).

### *Ammonium*

#### *Nonpoint Source*

- Natural: bacteria produce ammonia during decomposition of proteins and other nitrogenous organic substances in the water and benthic sediments. Animal and human excrement may contribute an insignificant amount of ammonia[8].
- Agricultural: the primary agricultural sources include accidental releases of ammonia-rich fertilizer during transport (because of vehicle accident, faulty hose connections, and human error); and livestock waste (from barnyards, feedlots, pastures, and rangeland).
- Residential and urban: household use of ammonia-containing cleaning products and improper disposal of ammonia products may contribute to nonpoint pollution.
- Other: evidence suggests that the atmospheric deposition of nitrogen (directly and via rainfall) constitutes a large portion of total nitrogenous inputs to estuarine and marine systems and a somewhat lesser portion of total nitrogen inputs to freshwater systems. Ammonia in the atmosphere is derived from combustion processes such as domestic heating, burning of municipal waste, and internal-combustion engines.

#### *Point Source*

The following industrial processes produce ammonia emissions and effluent: conversion of coal to coke in coke plants, metallurgic operations, ceramic production, strip mining, chemical synthesis (nitric acid, synthetic monomers, and plastics), waste gas treatment, sewage treatment plants, ammonium nitrate explosive production, production of refrigeration equipment, production of household cleaners, oil refineries, and food processing[8].

## METHODS

### Modeling System MIKE 11

The modeling system for rivers in MIKE 11 has been developed by VKI Water Quality Institute[9]. Its water quality module deals with the basic aspects of river water quality in areas influenced by human activities (e.g., oxygen depletion and ammonia levels as a result of organic matter loading). The water quality module solves a system of coupled differential equations describing the most important physical, chemical, and biological interactions in the river.

Concentrations of ammonium ($NH_4$-N) and nitrate ($NO_3$-N) are calculated in MIKE 11. Nitrogen is modeled according to two important forms of appearance: a nonoxidized form (ammonium) and an oxidized form (nitrate). Nitrification and denitrification have an influence on their concentrations. These processes are described with process velocities of first order (dC/dt-C), and the dependence on temperature with Arrhenius-terms ln(dC/dt)-T, with T the temperature of the river water. This way of presenting the processes is called macroscopic because it tries to represent the way they are observed macroscopically with equations. The different processes on a microscopic scale that form the basis of the macroscopic observation are thus not considered[10].

The equations describing the ammonium and nitrate reactions are:

$$\frac{d(NH_4 - N)}{dt} = -k_{nitr}(NH_4 - N)\theta_{nitr}^{T-20} + y_{deg\,rad}BOD\theta_{deg\,rad}^{T-20} - y_{resp}(p_h - r_s)$$

$$\frac{d(NO_3 - N)}{dt} = k_{nitr}(NH_4 - N)\theta_{nitr}^{T-20} - k_{denitr}(NO_3 - N)\theta_{denitr}^{T-20}$$

where $NH_4$-N = ammonium concentration, $NO_3$-N = nitrate concentration, BOD = biochemical oxygen demand, $y_{degrad}$ = nitrogen content in organic matter (0.29 mg N/mg BOD), $k_{degrad}$ = degradation rate for organic matter at 20°C (0.5/day), $k_{nitr}$ = nitrification rate at 20°C (0.54/day), $y_{nitr}$ = yield factor describing the amount of oxygen used at nitrification (4.47 mg $O_2$/mg N), $\theta_{degrad}$ = Arrhenius temperature coefficient of the degradation process (1.024), $\theta_{nitr}$ = Arrhenius temperature coefficient of the nitrification process (1.13), T = water temperature (°C), $y_{resp}$ = nitrogen release/uptake during respiration/photosynthesis (0.066 mg N/mg $O_2$), $k_{denitr}$ = denitrification rate (1/day), $\theta_{denitr}$ = Arrhenius temperature coefficient of the denitrification process (1.16), $r_s$ = respiration rate at 20°C (3 mg $O_2$/l/day), and $p_h$ = actual production of oxygen (3.5 mg $O_2$/l/day for the maximum rate).

The main processes affecting the ammonium concentration are nitrification, photosynthesis, and respiration. Also the degradation of BOD is a source of nutrients. The reactions influencing the nitrate concentration are nitrification and denitrification. The nitrification decay coefficient was calibrated based on the difference between detailed modeling results of the leached $NO_3$-N results (as input for MIKE 11, see section "Input Data and Models" below) and MIKE 11 results using the default value. As shown in Fig. 2, it is clear that the default value for this coefficient is quite high as the $NO_3$-N concentrations are increased due to the high speed of the nitrification process velocity. Therefore the value was reduced from 1.54 to 0.54, which also gave the best fit with the emission measurements for the $NO_3$-N results. For all other parameters, calibration of the water quality model parameters (process rates and Arrhenius terms) could not be performed because of the limited time resolution of the available emission measurements; therefore the default parameter values are used[9].

### Conceptual Simplified Model

The significance of different water quality processes varies depending on the case study considered. Because of the large limitations in the available data, only the most important processes are considered. To evaluate the different water quality processes, a sensitivity analysis is carried out. Based on the results, a simplified river water quality model is set up. To calibrate a simplified model to the more detailed MIKE 11 model, two simulations are performed with the MIKE 11 model. In the first simulation, only the advection-dispersion processes are considered while in the second simulation, the water quality processes are also included. By comparing the results of the first and the second simulation, concentration reduction factors are calibrated using the procedure followed by Willems[11].

### Sensitivity Analysis of Water Quality Processes

The aim of the sensitivity analysis is to estimate the rate of change in the output of a model with respect to changes in model inputs. Such knowledge is important for (1) evaluating the applicability of the model, (2) determining parameters for which it is important to have more accurate values, and (3) understanding the behavior of the system being modeled. A sensitivity analysis is carried out for the different water quality processes and parameters to assess the most sensitive processes and parameters for the purpose of river quality management and monitoring plans.

A comparison is made between the simulation results using default parameter values and a simulation in which the parameter values are drastically changed. The change in the simulation results is presented by the concentration reduction factor f, which is plotted for all modeled pollutants. Based on the results, the processes that affect the results in a significant way are considered in the conceptual simplified model. The processes examined by the sensitivity analysis are: nitrification, denitrification, BOD decay, photosynthesis, and respiration. Examples of studied sensitivities are presented in Fig. 3. They are derived for the case study of the Molenbeek River in Belgium located upstream of the village of Erpe-Mere. The hydrological and hydraulic results have been shown earlier in Radwan et al.[12,13].

For the Molenbeek River case study, the concentration reduction factor mainly depends on the nitrification and the denitrification processes. For $NH_4$-N, the concentration reduction factor mainly depends on the nitrification and BOD decay pro-

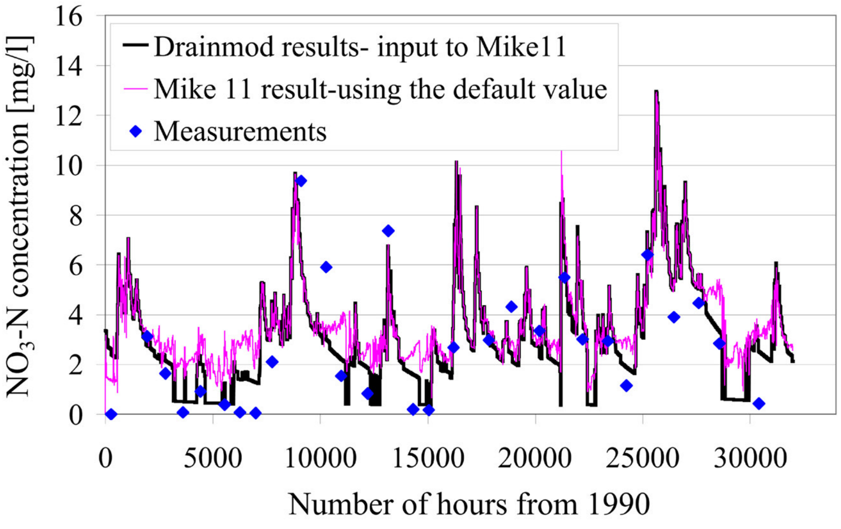

**FIGURE 2.** Effect of nitrification process on $NO_3$-N concentration.

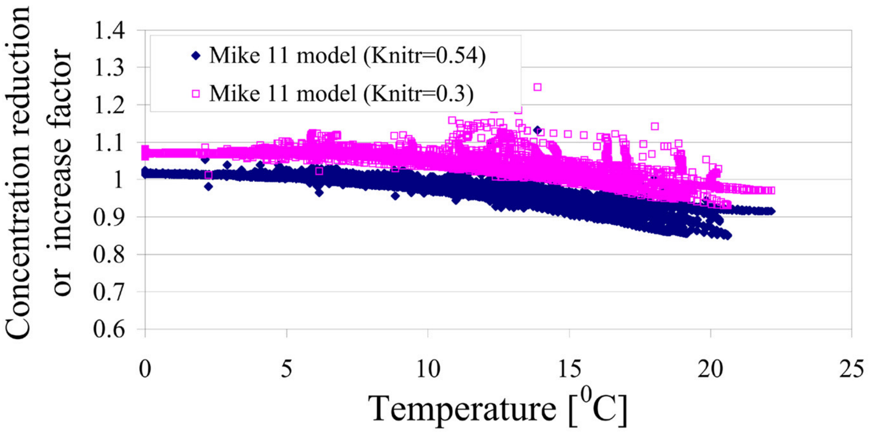

**FIGURE 3.** Influence of an increase in ammonium degradation (nitrification) rate on the concentration reduction factor for ammonium.

cesses. By considering only these processes, the following simplified river model is derived:

$$\frac{d(NH_4-N)}{dt} = -k_{nitr}(NH_4-N)\theta_{nitr}^{T-20} + y_{deg\,rad}k_{deg\,rad}BOD\theta_{deg\,rad}^{T-20}$$

$$\frac{d(NO_3-N)}{dt} = k_{nitr}(NH_4-N)\theta_{nitr}^{T-20} - k_{denitr}(NO_3-N)\theta_{denitr}^{T-20}$$

### Input Data and Models

Along the Molenbeek River, the major sources of pollution are agricultural drainage and industrial and domestic wastewater. Agricultural activities in the catchment are about 76% of the total area. To estimate nitrate leaching, a DRAINMOD-N model[14] is used. A detailed description of each functional relationship and a model application in the Netherlands and Flanders is given by El-Sadek et al.[15]. Its application for the Molenbeek River is given by Radwan et al.[13]. The climates, soil, crop, and crop management data required as input for the water and nitrate transport modeling were collected. The distributions over the catchment of the model input and simulation results were presented graphically using GIS. Combination of soil type and the main agricultural land units resulted in 32 scenarios—the result of 8 soil types and 4 main field crops. The soil-land use map was overlaid by the parcel map and for each parcel the climate, soil, crop conditions, and nitrogen application depth were determined and used as input for the DRAINMOD model. The leaching period from January 1, 1990 to December 31, 1997 was simulated using the measured $NO_3$-N concentrations on December 16, 1989 as the initial condition. The simulation results are used as input for MIKE 11. Along the Molenbeek River there are 21 sewerage outlet pipes that receive domestic wastes. For each point the total population discharging to it was calculated[16], and then the concentration of rough effluent was evaluated by assuming concentrations of ammonia equal to 10 g per capita per day[17]. The industrial wastes are considered one of the main sources of water pollution because of their toxic chemicals and organic loading. In the studied catchment there is one factory located at 10.990 km. The pollution load for different water quality parameters is estimated on the basis of measurements of the Flemish Environmental Agency (VMM) and assumed constant in time.

### RESULTS AND DISCUSSION

The MIKE 11 model and the conceptual simplified model are simulated for a period of 8 years. Observed flows, rain, and temperature hourly data for the period 1990 to 1997 were hereby used. The calculations were performed on an hourly basis. The simulation results for 2 years (Fig. 4 and Fig. 5) indicate that the general performance of the two models is satisfactory after a comparison made with the measurements. Regarding the simulated water quality state, it is concluded that the standards are not met for $NH_4$-N. This problem is mainly encountered in summer as a consequence of the combination of low discharges and pollutant loads ($NH_4$-N). During the periods with high discharges, the water quality indeed meets the standards. The comparison of the two models shows that the conceptual simplified model behaves similar to the detailed MIKE 11 model.

Two aspects of the adequacy of the model results are highlighted; namely accuracy and model speed. Model accuracy is defined as the difference between the modeled and observed values. Model speed is considered very important in order to perform long-term simulations and to analyze the river water quality state in a statistical way. It is also important if one wants to perform an uncertainty analysis. Indeed, in such analysis, many computer runs are needed. According to the speed, the conceptual simplified model is much faster than MIKE 11 model. To run a 1-year simulation with MIKE 11 model, 1 h is needed. But with the conceptual simplified model, only 2 s are needed. The conceptual simplified model is 1800 times faster. The accuracy of the two models is studied as the difference between the simulated value and reality. The measurement frequency is not very high, once a month. The standard deviation of the residuals between the measurements and the average simulated values of the same measurement day is calculated and results are presented in Table 1. From these results, it can be concluded that the two models have similar accuracy.

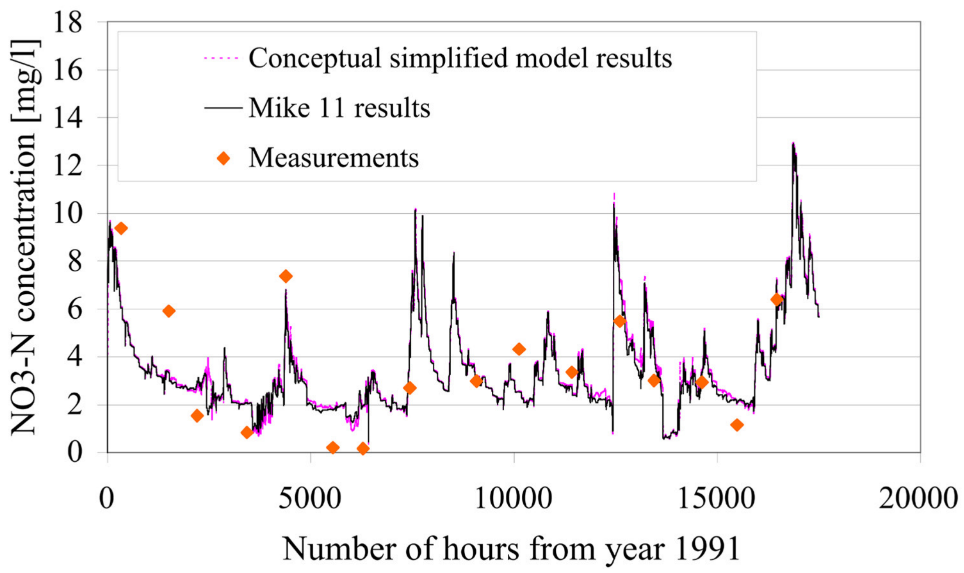

**FIGURE 4.** Modeled $NO_3$-N concentrations with both models against the measurements.

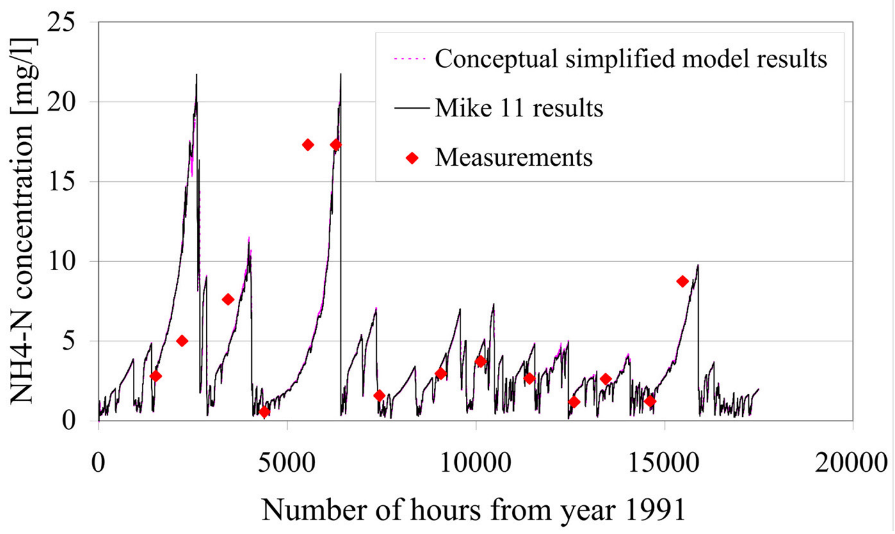

**FIGURE 5.** Modeled $NH_4$-N concentrations with both models against the measurements.

**TABLE 1**
**Comparison of the Standard Deviation for both the MIKE 11and the Conceptual Simplified Model**

| | The Standard Deviation | |
|---|---|---|
| **Modeled Parameter** | **MIKE 11** | **Conceptual Simplified Model** |
| $NO_3$-N | 1.32 | 1.3 |
| $NH_4$-N | 1.45 | 1.44 |

### CONCLUSION

To model catchment surface water quality, different model types are available. They vary from detailed physically based models to simplified conceptual and empirical models. The most appropriate model type for a certain application depends on the project objectives and the data availability. Nitrogen sources (agricultural leaching and domestic point sources) and modeling are presented in this paper. Specifically concentrations of ammonium ($NH_4$-N) and nitrate ($NO_3$-N) are modeled (using two models) with different levels of complexity. The calibration was done on the basis of available measured water quality data. To the detailed model a more simplified model was calibrated with the objective to more easily yield long-term simulation results, which can be used in a statistical analysis. Two aspects of the adequacy of model results are highlighted; namely, accuracy and model speed. The conceptual simplified model is 1800 times faster than the MIKE 11 model. Moreover the two models have almost the same accuracy. Therefore, the conceptual simplified model is recommended for long-term simulations as it includes only the most important and sensitive processes. In addition to this, as less input data are needed to run the simplified model, the total uncertainty of the results is smaller because the nonsensitive processes (with their input uncertainty) are excluded. Finally the detailed models are recommended for short-term simulations unless there are enough data for model input and model parameters.

| | | |
|---|---|---|
| Received: | July | 27, 2001 |
| Revised: | October | 5, 2001 |
| Accepted: | November | 1, 2001 |
| Published: | November | 27, 2001 |
